# Rho kinase inhibitor Y-27632 promotes the differentiation of human bone marrow mesenchymal stem cells into keratinocyte-like cells in xeno-free conditioned medium

**DOI:** 10.1186/s13287-015-0008-2

**Published:** 2015-03-11

**Authors:** Zhenzhen Li, Shichao Han, Xingqin Wang, Fu Han, Xiongxiang Zhu, Zhao Zheng, Hongtao Wang, Qin Zhou, Yunchuan Wang, Linlin Su, Jihong Shi, Chaowu Tang, Dahai Hu

**Affiliations:** Department of Burns and Cutaneous Surgery, Xijing Hospital, Fourth Military Medical University, No. 127 West Changle Road, Xi’an, 710032 Shaanxi China; Department of Neurosurgery, Tangdu Hospital, Fourth Military Medical University, No. 1 Xinsi Road, Xi’an, 710038 Shaanxi China

## Abstract

**Introduction:**

Bone marrow mesenchymal stem cells (BMSCs), which have the ability to self-renew and to differentiate into multiple cell types, have recently become a novel strategy for cell-based therapies. The differentiation of BMSCs into keratinocytes may be beneficial for patients with burns, disease, or trauma. However, the currently available cells are exposed to animal materials during their cultivation and induction. These xeno-contaminations severely limit their clinical outcomes. Previous studies have shown that the Rho kinase (ROCK) inhibitor Y-27632 can promote induction efficiency and regulate the self-renewal and differentiation of stem cells. In the present study, we attempted to establish a xeno-free system for the differentiation of BMSCs into keratinocytes and to investigate whether Y-27632 can facilitate this differentiation.

**Methods:**

BMSCs isolated from patients were cultured by using a xeno-free system and characterised by using flow cytometric analysis and adipogenic and osteogenic differentiation assays. Human primary keratinocytes were also isolated from patients. Then, the morphology, population doubling time, and β-galactosidase staining level of these cells were evaluated in the presence or absence of Y-27632 to determine the effects of Y-27632 on the state of the keratinocytes. Keratinocyte-like cells (KLCs) were detected at different time points by immunocytofluorescence analysis. Moreover, the efficiency of BMSC differentiation under different conditions was measured by quantitative real-time-polymerase chain reaction (RT-PCR) and Western blot analyses.

**Results:**

The ROCK inhibitor Y-27632 promoted the proliferation and lifespan of human primary keratinocytes. In addition, we showed that keratinocyte-specific markers could be detected in BMSCs cultured in a xeno-free system using keratinocyte-conditioned medium (KCM) independent of the presence of Y-27632. However, the efficiency of the differentiation of BMSCs into KLCs was significantly higher in the presence of Y-27632 using immunofluorescence, quantitative RT-PCR, and Western blot analyses.

**Conclusions:**

This study demonstrated that Y-27632 could promote the proliferation and survival of human primary keratinocytes in a xeno-free culture system. In addition, we found that BMSCs have the ability to differentiate into KLCs in KCM and that Y-27632 can facilitate this differentiation. Our results suggest that BMSCs are capable of differentiating into KLCs *in vitro* and that the ROCK pathway may play a critical role in this process.

## Introduction

Skin defects are a severe problem for patients suffering from scar resection, burn injury, trauma, or chronic ulcers after systemic diseases. Currently, the primary cure for most skin defects is the use of skin grafting. However, the current supply of available skin grafts is far less than the tremendous demand. Consequently, the development of additional methods to provide enough skin is urgently required.

Compared with autoplastic and allograft skin, cell-based therapies are a promising area of research because cells are easier to obtain and to expand and have richer resources; thus, cell-based therapies may benefit patients in need of skin replacement because of burns, disease, or trauma. Recently, advances in stem cell techniques have provided novel strategies and methods for the therapy of skin lesions. Stem cells are ideal candidate cells because of their ability to self-renew and to generate committed progenitors. Among the various stem cells that have been identified thus far, adult stem cells are the most suitable cells not only because of their skin healing and regenerative capabilities but also because of ethical and moral reasons. Of all the adult stem cell types, mesenchymal stem cells (MSCs) are of great interest because of their easy isolation, multipotency, and high proliferative potential *in vitro* [1]. Additionally, from a clinical point of view, the use of bone marrow-derived MSCs (BMSCs) in cell therapy is extremely convenient for patients with skin defects because these cells can be harvested easily from patients during bone marrow aspiration and then expanded in culture. Indeed, previous studies have reported that BMSCs can not only act in the haematopoietic system but also migrate into damaged tissues and organs and inductively differentiate into corresponding cells [2-5]. Furthermore, BMSCs have gained great interest in regenerative medicine, and several preclinical models and clinical trials have demonstrated their safety and efficiency in various clinical applications [6]. Moreover, human BMSCs in particular are capable of differentiating into epithelial-like cells [7]. Together, these findings strongly indicate the great potential for the clinical application of BMSCs in skin regeneration.

Currently, the standard practice of culturing BMSCs is based on supplementing the basal medium with foetal bovine serum (FBS) and on dissociating the cells with porcine-derived trypsin. The use of these two ingredients increases the potential risk of graft rejection [8,9] and the transfer of non-human pathogens. Hence, the development of a system of BMSC expansion under xeno-free, serum-free conditions is necessary for the improved clinical application of BMSCs. MesenCult-XF medium, which is a defined serum- and xeno-free medium, has been used previously to culture MSCs [10-12]. Cells cultured in MesenCult-XF medium showed a similar isolation efficiency and exhibited typical BMSC characteristics compared with those cultured in conventional serum-containing medium [11]. In addition, the cell dissociation enzyme TrypLE Select, which is free of any animal-derived components, can be used for the dissociation of cultured MSCs instead of porcine-derived trypsin to avoid xeno-contamination. Recently, several groups demonstrated the isolation of MSCs from various tissue sources under xeno-free, serum-free conditions [10-12]. Therefore, owing to the efficiency and the great advantage of using xeno-free medium, MesenCult-XF medium and TrypLE Select were used to culture BMSCs in this study.

Changes in the cellular microenvironment are considered the key factors for initiating differentiation [13,14]. Conditioned medium derived from keratinocyte culture supernatants contains secreted growth factors and small molecules that are able to activate MSC differentiation [14]. Currently, the optimal condition for culturing primary keratinocytes consists of feeder cells and F medium [15,16]. However, this condition inherently produces xeno-contamination caused by the feeder cells of animal origin and by the presence of animal proteins from the FBS and other medium supplements derived from mouse fibroblasts; this contamination severely limits the potential application of these cultured cells in clinical practice. Thus, a defined keratinocyte serum-free medium (DKSFM) was optimised to obtain xeno-free medium for BMSC differentiation and for supporting the growth and expansion of primary and secondary human keratinocytes without the use of fibroblast feeder layers. Considering this system, we attempted to establish a xeno-free system in the present study for the culture of keratinocytes and for the subsequent differentiation of BMSCs into keratinocytes.

Y-27632 is an inhibitor of Rho kinase (ROCK), which regulates cellular growth, adhesion, migration, metabolism, and apoptosis by controlling actin cytoskeleton assembly and cell contractions [2,17]. Previous studies have demonstrated that Y-27632 is useful in the generation of human-induced pluripotent stem cells by introducing reprogramming factors [3-5] and can promote the induction efficiency of these stem cells. Furthermore, supplementary Y-27632 can regulate the ability of stem cells to self-renew and differentiate into derivatives of all three germ layers [18-20]. More importantly, Y-27632 was shown to greatly increase the long-term proliferation of primary human keratinocytes and, unexpectedly, to enable these cells to efficiently bypass senescence and become immortal without a detectable cell crisis [15,16]. Accordingly, in addition to the establishment of a xeno-free system for the culture of keratinocytes and for the differentiation of BMSCs into keratinocytes, we further investigated whether Y-27632 could also promote the proliferation of primary human keratinocytes under xeno-free conditions and affect the differentiation of BMSCs into keratinocytes. We found that continuous ROCK inhibition promoted the proliferation of keratinocytes and inhibited cellular senescence for at least 40 population doublings in the DKSFM. We also showed that BMSCs can differentiate into keratinocyte-like cells (KLCs) in our xeno-free conditioned medium and that BMSCs acquired a higher efficiency of differentiation in the presence of Y-27632 compared with the control group.

## Methods

### Cell culture

All human tissues were obtained from patients (mean age of 30 years) at Xijing Hospital (Xi’an, China). Before the experiment, all patients were informed about the purpose and procedures of the study and voluntarily agreed to provide tissue. Written consent was obtained from all participants, and all protocols were approved by the Ethics Committee of Xijing Hospital, which is affiliated with the Fourth Military Medical University. For BMSC isolation, bone marrow aspirates were taken from the iliac crest of patients, and the mononuclear cell (MNC) fraction from the bone marrow was isolated by using density gradient centrifugation. Then, isolated MNCs were seeded in 25-cm^2^ tissue culture flasks at 37°C and 5% CO_2_ and grown in MesenCult-XF medium (Stemcell Technologies, Vancouver, BC, Canada), which is a defined serum- and xeno-free medium. MesenCult-SF (Stemcell Technologies) attachment substrate was used to pre-coat the culture plates for 2 hours at room temperature. The medium was changed every 2 days until the cells reached confluency. The cells were dissociated with TrypLE Select (Gibco, Grand Island, NY, USA) and used for experiments at passages 4 to 7. Primary human keratinocytes were derived from patients (age of 20 ± 10 years) undergoing skin grafting procedures. Briefly, the epidermal layer of human keratinocytes was separated from the dermis and placed into a sterile 15-mL conical tube containing 2 mL of 0.05% trypsin-EDTA. The cells were incubated at 37°C for approximately 15 minutes, during which time the cells were triturated by using a 2-mL pipette every 2 to 3 minutes to aid in cell dissociation. The cells were centrifuged at 180 *g* for 7 minutes at room temperature. After resuspension, the primary keratinocytes were seeded in 75-cm^2^ tissue culture flasks at a density of approximately 3 × 10^6^ cells per flask in 15 mL of DKSFM with the addition of DKSFM growth supplement, which eliminated the requirement for bovine pituitary extract (Gibco), and grown in the presence or absence of 10 μM of the ROCK inhibitor Y-27632 (Sigma-Aldrich, St. Louis, MO, USA) and 1% antibiotics (penicillin/streptomycin 1,00 U). The medium was changed every 2 days until the cells reached confluency, and the keratinocytes were cultured continuously until use.

### Flow cytometric analysis

The expression of cell surface markers on BMSCs cultured in the xeno-free medium at passage 5 was assessed by using flow cytometry. The BMSCs were incubated with primary antibodies for 30 minutes at 4°C after washing twice with phosphate-buffered saline (PBS). The primary antibodies used were as follows: anti-human CD29-PE, anti-human CD31-FITC, anti-human CD34-FITC, anti-human CD45-FITC, anti-human CD90-FITC, and anti-human CD105-PE. All antibodies were purchased from BD Pharmingen (San Diego, CA, USA) and used in accordance with the instructions of the manufacturer. After incubation, the cells were washed twice with PBS and analysed by using a flow cytometer (BD FACSAria™ III system; BD Pharmingen).

### Differentiation of bone marrow mesenchymal stem cells *in vitro*

Human BMSCs at passage 5 were plated in six-well tissue culture plates that were pre-coated with a 0.1% gelatin solution (Cyagen Bioscience, Inc., Guangzhou, China) for 2 hours at a density of 3 × 10^4^ cells/cm^2^. After 24 hours, the BMSCs were induced with osteogenic and adipogenic differentiation media (Cyagen Bioscience, Inc.) for 2 and 3 weeks, respectively. Then, the induced cells were fixed with 4% paraformaldehyde in PBS (pH 7.4) for 15 minutes at room temperature, and the cells were stained separately with Alizarin Red S for 5 minutes and Oil Red O for 30 minutes at room temperature. Then, the cells were washed with PBS, and images were captured by using an Olympus IX71 light microscope (Olympus, Tokyo, Japan).

### Estimation of population doubling rate

The number of keratinocytes harvested at each passage was determined, and the population doubling was calculated as follows: 3.32(log[number of cells harvested/number of cells seeded]) [21].

### Beta-galactosidase staining

Keratinocytes were plated in 35-cm^2^ wells for 24 hours before the staining procedure. The detection of cellular senescence was performed by using a Senescence β-Galactosidase Staining Kit (Beyotime, Shanghai, China) in accordance with the instructions of the manufacturer. Briefly, the cells were washed three times with PBS, fixed for 15 minutes in stain fixative at room temperature, and washed three times with PBS. Then, the cells were incubated with the β-galactosidase staining solution at 37°C (no CO_2_) overnight. Images were captured by using a light microscope (Olympus IX71). The β-galactosidase-positive cells, which developed blue granules, were considered senescent.

### Differentiation of human bone marrow mesenchymal stem cells into keratinocyte-like cells

Keratinocyte-conditioned medium (KCM) was used to differentiate BMSCs into KLCs. Briefly, KCM was derived from keratinocytes by collecting the medium from cultured human keratinocytes at passages 2 to 5 with (KCM2) or without (KCM1) 10 μM of the ROCK inhibitor Y-27632 at 60% to 70% confluence every day. The collected media were centrifuged at 1,000 revolutions per minute (rpm) for 5 minutes to remove cells, filtered through a 0.4-μm filter, and diluted with an equal volume of MesenCult-XF medium before use. Beginning at 24 hours after plating, BMSCs at passages 4 to 7 were treated daily with the diluted media for up to 21 days. To assess the differentiation of BMSCs into KLCs, BMSCs were harvested for either RNA extraction or protein extraction, and the expression of keratinocyte-specific genes and proteins was analysed by using real-time-polymerase chain reaction (RT-PCR) and Western blot analyses, respectively.

### Quantitative RT-PCR analysis

Total RNA from the cells was extracted by using TRIzol Reagent (Invitrogen, Carlsbad, CA, USA) in accordance with the protocol of the manufacturer. The OD260/280 ratio of the RNA samples was measured, and the samples with a ratio of 2.0 were used for reverse transcription. In total, 0.5 mg RNA was reverse-transcribed by using PrimeScript RT Master Mix (TaKaRa, Dalian, China). The reactions were performed at 37°C for 15 minutes and then 85°C for 5 seconds. Quantitative RT-PCR analyses were performed in triplicate by using SYBR Green PCR Master Mix (TaKaRa) and detected by using a Bio-Rad iQ5 Multicolor Real-Time PCR Detection System (Bio-Rad, Hercules, CA, USA). The data were normalised to β-actin, and the comparative cycle threshold (Ct) method (2^ΔΔCT^) was used to calculate the relative quantity of target mRNAs. The primer sequences used are provided in Table 1.

**Table 1:** **Names and sequences of primers used for quantitative real-time-polymerase chain reaction**

**Name**	**Species**	**Sense**	**Antisense**
Cytokeratin-5	Human	CATGCAGGACCTGGTGGAAG	CACAAACTCATTCTCAGCAGTGGTA
Cytokeratin-14	Human	GCGGCCTGTCTGTCTCAT	CCACCAGAAGCCCATCAC
Involucrin	Human	TCCTCCAGTCAATACCCATCAG	CAGCAGTCATGTGCTTTTCCT
Stratifin	Human	GCCAAGACCACTTTCGACGAG	CATGATGAGGGTGCTGTCTTTGTAG
Filaggrin	Human	GTGGCAGTCCTCACAGTTCTAGTTC	CCATAGCTGCCATGTCTCCAA
β-actin	Human	TGGCACCCAGCACAATGAA	CTAAGTCATAGTCCGCCTAGAAGCA

### Immunocytofluorescence analysis

For immunocytofluorescence analysis, cells cultured on coverslips were placed in 24-well plates and fixed in 4% paraformaldehyde in PBS (pH 7.4) for 15 minutes at room temperature. The fixed cells were washed twice with ice-cold PBS and then incubated with PBS containing 0.1% Triton X-100 for 10 minutes, followed by three washes with PBS. After blocking in bovine serum albumin (BSA) blocking buffer (1% BSA and 0.1% Tween 20 in PBS) for 1 hour, the cells were incubated in BSA blocking buffer containing primary antibodies—anti-cytokeratin-5 monoclonal antibody (mAb) (1:200; Abcam, Cambridge, UK), anti-cytokeratin-14 mAb (1:200; Abcam), anti-involucrin mAb (1:200; GeneTex, San Antonio, TX, USA), and anti-stratifin polyclonal antibody (polyAb) (1:200; GeneTex)—in a humidified chamber overnight at 4°C. The cells were washed and then incubated in secondary antibodies—anti-mouse (1:100; Life Technologies, Carlsbad, CA, USA) or anti-rabbit (1:100; Life Technologies)—for 1 hour. Next, the fixed cells were incubated for 15 minutes with 4,6-diamidino-2-phenylindole (DAPI) (Zhongshanjinqiao, Beijing, China) for nuclear staining, and fluorescence micrographs were obtained by using an Olympus FSX100 fluorescence microscope.

### Western blot analysis

Cells were washed with ice-cold PBS, lysed with RIPA buffer (HEART Biological Technology Co. Ltd., Xi’an, China) on ice for 30 minutes, and centrifuged at 12,000 rpm for 10 minutes to obtain protein. The protein samples were mixed with 5 × SDS-PAGE loading buffer (HEART Biological Technology Co. Ltd.) and heated at 100°C for 10 minutes. In total, 40 μg of protein lysate was loaded onto SDS-PAGE (HEART Biological Technology Co. Ltd.) for Western blot analysis. The primary antibodies used for membrane incubation were as follows: anti-cytokeratin-5 mAb (1:1,000), anti-cytokeratin-14 mAb (1:70), anti-involucrin mAb (1:1,000), anti-stratifin polyAb (1:1,000), and anti-filaggrin polyAb (1:1,000). Anti-β-actin polyAb (1:500; Santa Cruz Biotechnology, Dallas, TX, USA) was used as a loading control. Then, immunoreactive proteins were visualised by using ECL Western blotting detection reagent (Millipore, Billerica, MA, USA) and detected by using MultiImage Light Cabinet Filter Positions (Alpha Innotech, San Leandro, CA, USA).

### Statistical analysis

All statistical analyses were calculated by using GraphPad Prism software (version 4.0; GraphPad Software, La Jolla, CA, USA). Each experiment was repeated at least three times, and the data are presented as the mean ± standard error of the mean. Statistical significance was determined by using Student’s *t* tests or one-way analysis of variance. A *P* value of less than 0.05 was considered statistically significant.

## Results

### Characterisation of xeno-free bone marrow mesenchymal stem cells

**Figure 1:**
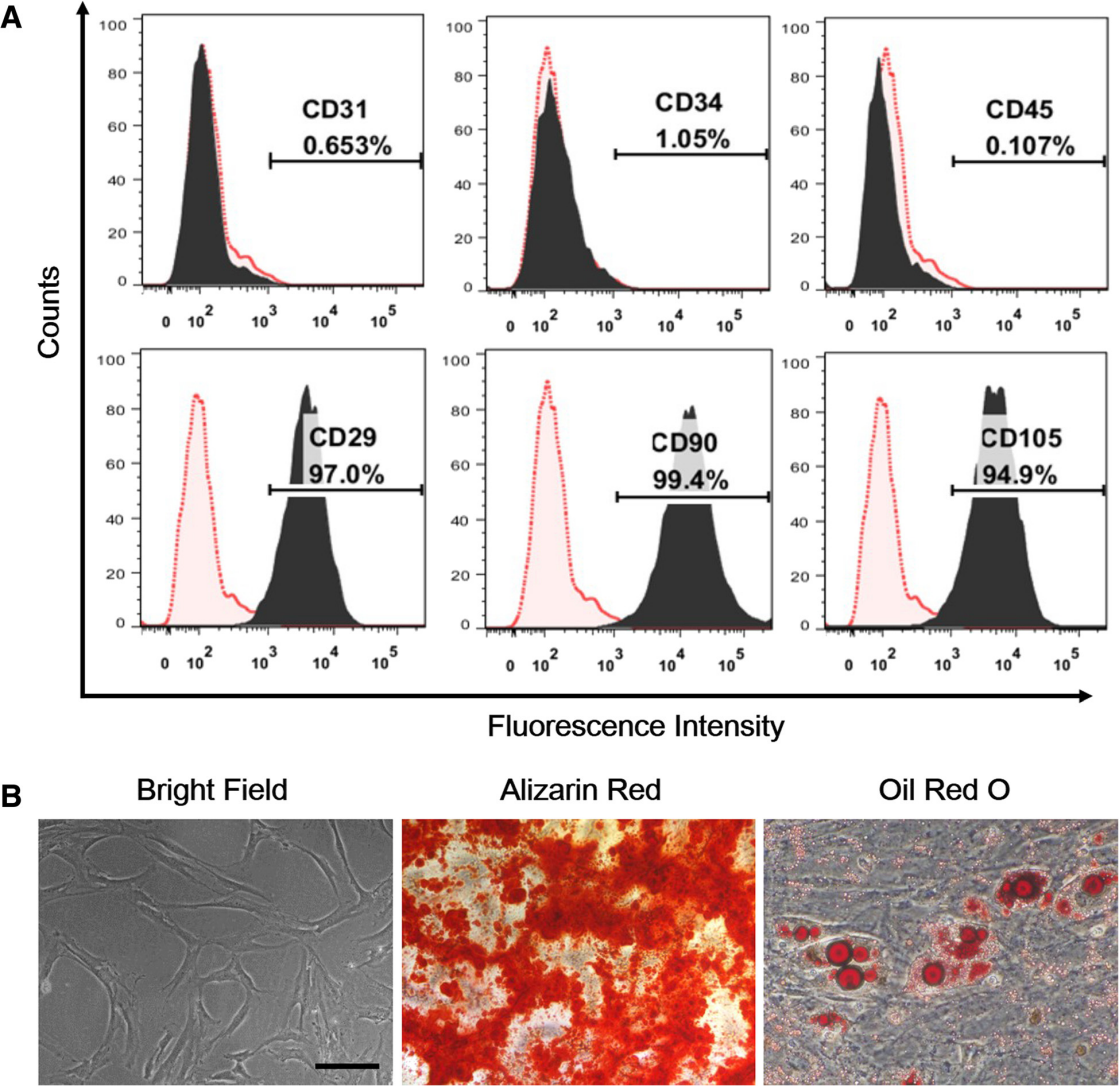
**Characterisation of xeno-free bone marrow mesenchymal stem cells (BMSCs)** ***in vitro*** **. (A)** Flow cytometry results show that xeno-free BMSCs were uniformly positive for the mesenchymal stem cell markers CD29, CD90, and CD105 but were negative for the haematopoietic markers CD31 and CD34 and for the leukocyte common antigen CD45. **(B)** Isolated BMSCs exhibited a spindle-like morphology under light field microscopy. Adipogenic and osteogenic differentiations were detected by Oil Red O staining and by Alizarin Red S staining, respectively, to confirm the multipotency of BMSCs. Scale bar: 50 μm.

Previous studies have found no significant differences between cells grown in xeno-free conditions and cells grown in FBS-containing medium [10-12]. In this study, isolated BMSCs were cultured in MesenCult-XF medium, which is a defined serum- and xeno-free medium, to eliminate xeno-contamination throughout the culture process. Then, flow cytometric analysis of isolated BMSCs was performed at passage 5. As shown in Figure 1A, the xeno-free BMSCs expressed the MSC markers CD29, CD90, and CD105, whereas these cells were negative for the haematopoietic markers CD31 and CD34 and for the leukocyte common antigen CD45 [22]. Furthermore, the BMSCs exhibited a high rate of proliferation and a fibroblast-like morphology (Figure 1B). Next, we examined the multipotency of BMSCs from patients by using adipogenic and osteogenic differentiation assays. After being induced with adipogenic medium for 3 weeks, BMSCs had an adipocyte phenotype as confirmed by Oil Red O staining (Figure 1B). The cells were incubated in osteoblastogenic medium for 2 weeks and then stained with Alizarin Red S to examine the osteoblastogenic differentiation ability of BMSCs; the staining revealed the presence of calcium deposits (Figure 1B). Together, these results demonstrate that isolated BMSCs under xeno-free conditions exhibited typical MSC characteristics as previously reported [11].

### The effect of the Rho kinase inhibitor Y-27632 on primary adult keratinocytes

**Figure 2:**
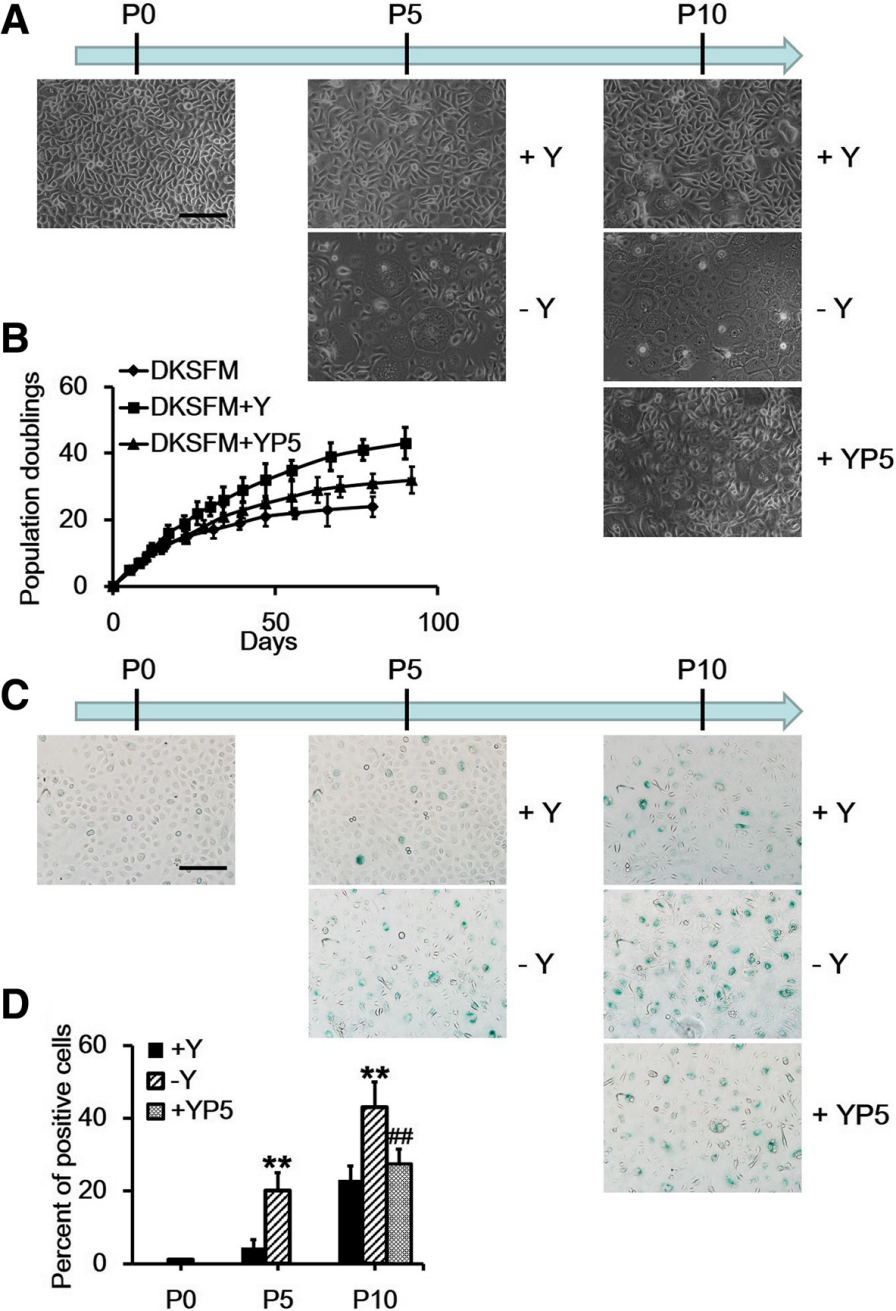
**Effect of Y-27632 on the culturing of keratinocytes. (A)** The morphology of keratinocytes from the three groups at the indicated time points (P0, P5, and P10). **(B)** The growth curves of keratinocytes with various treatments. **(C)** Beta-galactosidase staining of keratinocytes from the three groups at the indicated time points (P0, P5, and P10). **(D)** The average number of cells staining positive for β-galactosidase was divided by the average total number of cells (nuclei counts) and multiplied by 100 to obtain the percentage. Data are presented as the mean ± standard error of the mean. n = 3; scale bar: 50 μm. **P<0.01 keratinocytes without Y-27632 versus keratinocytes with Y-27632, ##P<0.01 keratinocytes without Y-27632 versus Y-27632 added to keratinocytes at passage 5. DKSFM, defined keratinocyte serum-free medium; DKSFM + Y, defined keratinocyte serum-free medium supplemented with Y-27632 at passage 0; DKSFM + YP5, defined keratinocyte serum-free medium supplemented with Y-27632 at passage 5; Y, Y-27632; YP5, Y-27632 added to keratinocytes at passage 5.

The ROCK inhibitor Y-27632 significantly increased the long-term proliferative capacity of primary human keratinocytes in a fibroblast feeder culture [15,16,23]. However, feeder cells derived from mouse fibroblasts are not suitable for clinical applications. Thus, we used DKSFM, which is a medium with no fibroblast feeder layers, as a xeno-free culture condition for culturing keratinocytes in the present study. Furthermore, the ROCK inhibitor Y-27632 (10 μM) was applied to determine whether continuous ROCK inhibition was required for the sustained proliferation of primary human keratinocytes cultured in DKSFM. For these experiments, Y-27632-treated cells were passaged at 80% to 90% confluence, and untreated cells were passaged until senescence. After treatment with Y-27632, primary keratinocytes can be continuously cultured for at least 10 passages with a healthy status. Y-27632 treatment inhibited cell senescence in the DKSFM for at least 40 population doublings, whereas only approximately 50% of the untreated cells escaped senescence at passage 5 (Figure 2A, B). The primary keratinocytes in the untreated control group at early passages were actively dividing and appeared small, cuboidal, and homogeneous. However, the cell morphology began to change at later passages, and the cells became flat and heterogeneous with an enlarged cytoplasmic volume. In addition, the proliferation of untreated cells gradually decreased with time (Figure 2A). By contrast, the majority of the cells in the Y-27632-treated group maintained a condition similar to that of early-passage cells even at passage 10 (Figure 2A). Y-27632 was added to keratinocytes at passage 5 when approximately 50% of the cells were already senescent to further determine the effect of Y-27632 on primary keratinocytes. Intriguingly, we found that Y-27632 markedly increased the rate of proliferation and successfully rescued the keratinocytes from further senescence (Figure 2A, B). β-Galactosidase staining was performed to detect cellular senescence to further confirm the effect of Y-27632 on the survival and proliferation of keratinocytes. As shown in Figure 2C and D, Y-27632 significantly decreased the number of β-galactosidase-positive cells when applied at either passage 0 or 5. These data suggest that inhibiting ROCK can promote keratinocyte proliferation and inhibit keratinocyte senescence when these cells are cultured in DKSFM, even when only half of their replicative cycles remained.

### Differentiation of bone marrow mesenchymal stem cells into keratinocyte-like cells under different conditions

**Figure 3:**
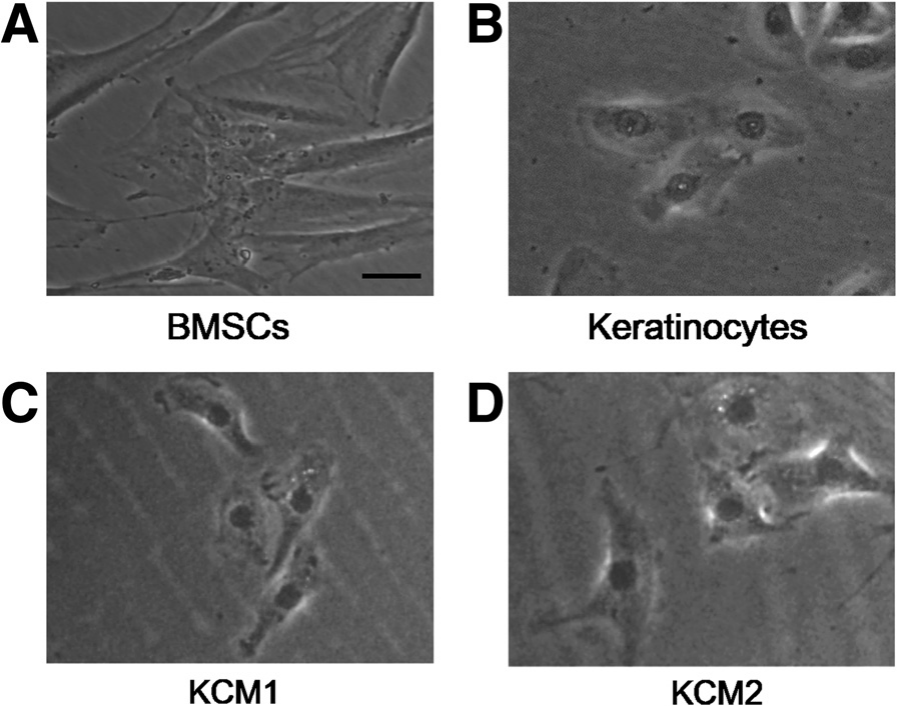
**Bone marrow mesenchymal stem cells (BMSCs) differentiated into keratinocyte-like cells after induction by keratinocyte-conditioned medium.** Evaluation of the cell morphology of BMSCs **(A)**, keratinocytes **(B)**, and KCM1 **(C)** and KCM2 **(D)** groups. Scale bar: 5 μm. KCM1, keratinocyte-conditioned medium collected from keratinocytes with no Y-27632 treatment; KCM2, keratinocyte-conditioned medium collected from keratinocytes treated with Y-27632.

Previous studies have shown that conditioned media contain secreted growth factors and small molecules that are able to activate BMSC differentiation [7,24]. Therefore, two types of KCMs were collected from cultured human keratinocytes at passages 2 to 5 in the presence (KCM2) or absence (KCM1) of Y-27632 pre-treatment (10 μM) to investigate whether BMSCs have the capacity to differentiate into KLCs under xeno-free conditions. KCM1 and KCM2 were collected daily at 60% to 70% confluence. Then, equal volumes of MesenCult-XF medium and either KCM1 or KCM2 were combined to culture the BMSCs. The morphology of cells in both groups changed gradually over time. After 2 weeks of treatment, approximately 30% of the Y-27632-treated BMSCs and approximately 20% of the untreated cells had changed from a fibroblast-like shape to a cobblestone-like shape (Figure 3), which was extremely similar to the morphology of keratinocytes. These results suggest that morphological changes were induced in BMSCs under both conditions.

### Keratinocyte-like cells express keratinocyte markers

**Figure 4:**
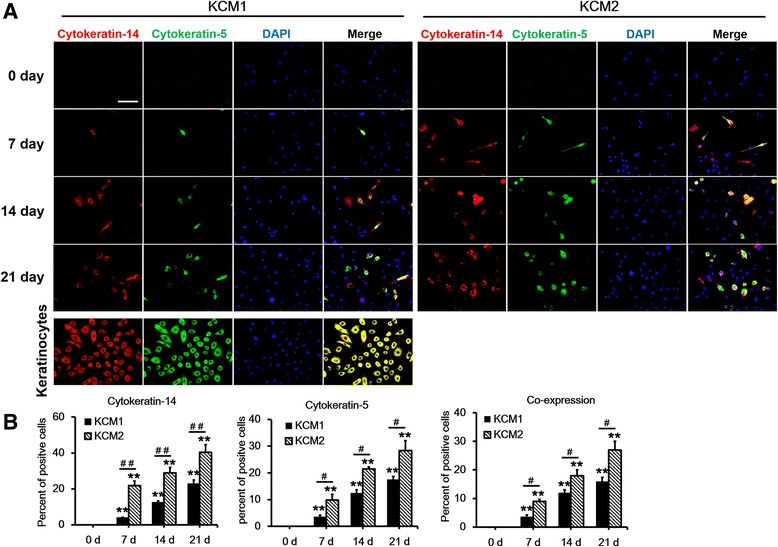
**Keratinocyte-conditioned medium induced the expression of the keratinocyte-specific markers cytokeratin-14 and cytokeratin-5. (A)** Bone marrow mesenchymal stem cells (BMSCs) from different induction media and time points (days 0, 7, 14, and 21) were co-labelled with cytokeratin-14 and cytokeratin-5. DAPI (4,6-diamidino-2-phenylindole) was used as a counterstain. Keratinocytes at passages 2 to 5 and BMSCs at passages 4 to 7 were used as positive and negative controls, respectively. Scale bar: 50 μm. **(B)** The average number of positively stained cells divided by the average total number of cells (nuclei counts) and multiplied by 100 to obtain the percentage. Data are presented as the mean ± standard error of the mean. n = 3 slides per time point. **P <0.01 compared with the relative value at day 0, ^#^ *P* <0.05, ^##^ *P* <0.01 KCM1 versus KCM2. KCM1, keratinocyte-conditioned medium collected from keratinocytes with no Y-27632 treatment; KCM2, keratinocyte-conditioned medium collected from keratinocytes treated with Y-27632.

A series of keratinocyte-specific markers were selected and evaluated by using immunofluorescence, quantitative RT-PCR, and Western blot analyses to further identify the altered cells. BMSCs were seeded on cover slides and treated with KCM1 or KCM2 for different periods (0, 7, 14, or 21 days) until the experiments were performed. Keratinocytes were used as positive controls. Interestingly, the keratinocyte markers cytokeratin-5 and cytokeratin-14 appeared in both groups as early as 7 days after treatment with either KCM1 or KCM2, and the percentage of positive cells increased gradually over time, with a maximum level occurring at 3 weeks (Figure 4A). Moreover, the ratio of positive cells in the Y-27632-containing medium (KCM2) was significantly higher than that in the control group (KCM1) at all observed periods (Figure 4). Accordingly, the detection of all subsequent markers was performed after 21 days of KCM treatment.

**Figure 5:**
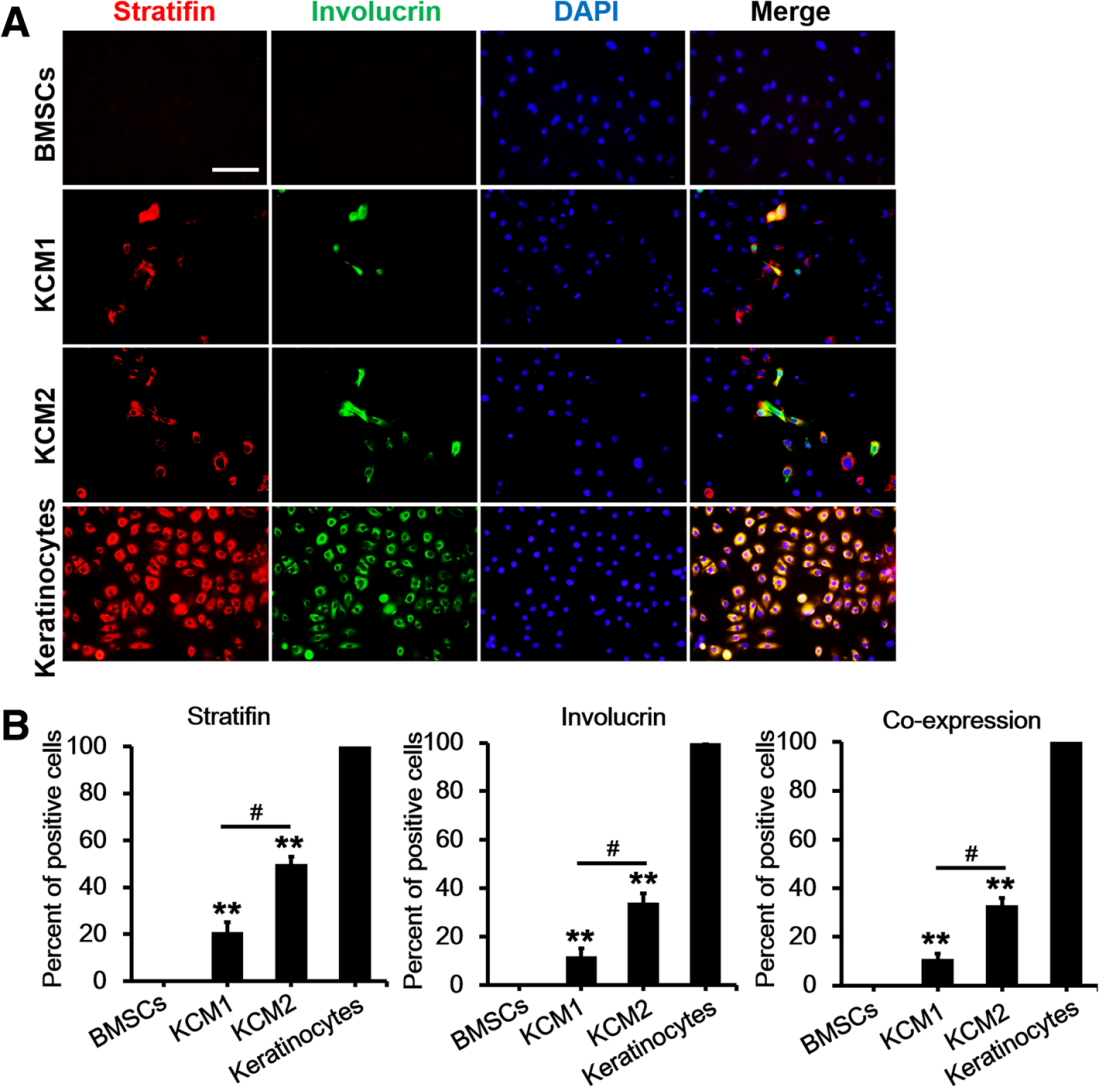
**Immunofluorescent analysis of stratifin and involucrin 21 days after the application of different keratinocyte-conditioned media. (A)** Bone marrow mesenchymal stem cells (BMSCs) were co-immunostained with stratifin and involucrin in two conditions. DAPI (4,6-diamidino-2-phenylindole) was used as a counterstain. Keratinocytes at passages 2 to 5 and BMSCs at passages 4 to 7 were used as positive and negative controls, respectively. Scale bar: 50 μm. **(B)** The average number of positively stained cells divided by the average total number of cells (nuclei counts) and multiplied by 100 to obtain the percentage. Data are presented as the mean ± standard error of the mean. n = 3 slides per group. **P <0.01 BMSCs versus KCM1/KCM2, ^#^ *P* <0.05 KCM1 versus KCM2. KCM1, keratinocyte-conditioned medium collected from keratinocytes with no Y-27632 treatment; KCM2, keratinocyte-conditioned medium collected from keratinocytes treated with Y-27632.

**Figure 6:**
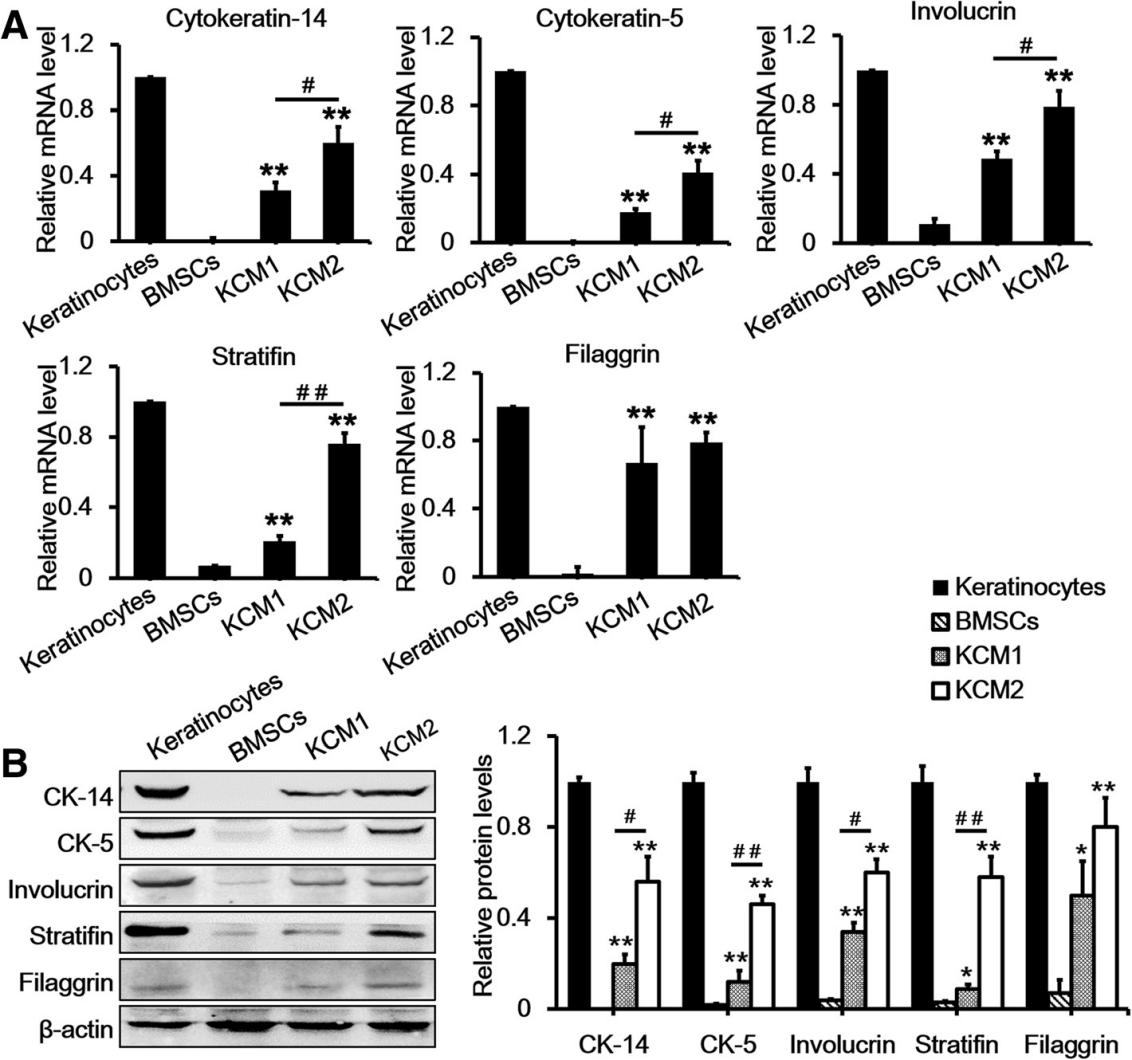
**Quantitative real-time-polymerase chain reaction and Western blot analysis of keratinocyte markers after a 21-day treatment with two different keratinocyte-conditioned media. (A)** mRNA expression of cytokeratin-5, cytokeratin-14, stratifin, involucrin, and filaggrin in bone marrow mesenchymal stem cells (BMSCs), keratinocytes, and KCM1 and KCM2 groups. Keratinocytes at passages 2 to 5 and BMSCs at passages 4 to 7 were used as positive and negative controls, respectively. Keratinocytes were normalised to 1 because these cells expressed all of the markers. **(B)** Western blot analysis of cytokeratin-5, cytokeratin-14, stratifin, involucrin, and filaggrin in BMSCs, keratinocytes, and KCM1 and KCM2 groups. Keratinocytes at passages 2 to 5 and BMSCs at passages 4 to 7 were used as positive and negative controls, respectively. Beta-actin was used as a loading control. Data are presented as the mean ± standard error of the mean. n = 3. *P <0.05, **P <0.01 BMSCs versus KCM1/KCM2, ^#^ *P* <0.05, ^##^ *P* <0.01 KCM1 versus KCM2. CK-5, cytokeratin-5; CK-14, cytokeratin-14; KCM1, keratinocyte-conditioned medium collected from keratinocytes with no Y-27632 treatment; KCM2, keratinocyte-conditioned medium collected from keratinocytes treated with Y-27632.

After 21 days of KCM treatment, co-immunostaining for stratifin and involucrin revealed results that were consistent with the cytokeratin staining results (Figure 5). In addition, the quantitative RT-PCR results showed that both the KCM1- and KCM2-treated BMSCs expressed higher levels of keratinocyte-specific markers (that is, cytokeratin-14, cytokeratin-5, stratifin, filaggrin, and involucrin) compared with untreated BMSCs. Furthermore, each of these markers except filaggrin was significantly higher in Y-27632-treated cells than in untreated cells (Figure 6A). Proteins were extracted from BMSCs, keratinocytes, and KCM-treated BMSCs and analysed for the same keratinocyte-specific markers by using Western blotting to further confirm the mRNA results. As shown in Figure 6B, untreated BMSCs had barely detectable levels of these markers, whereas both of the KCM-treated BMSC groups expressed these markers. Consistent with the mRNA levels, the expression levels of the selected markers were much higher in KCM2-treated BMSCs compared with those in the KCM1 group. Overall, the induced BMSCs demonstrated gene and protein expression profiles similar to keratinocytes, and the efficiency of differentiation was significantly increased by Y-27632 treatment.

### Y-27632 acts directly or indirectly on bone marrow mesenchymal stem cells to promote their differentiation into keratinocyte-like cells

**Figure 7:**
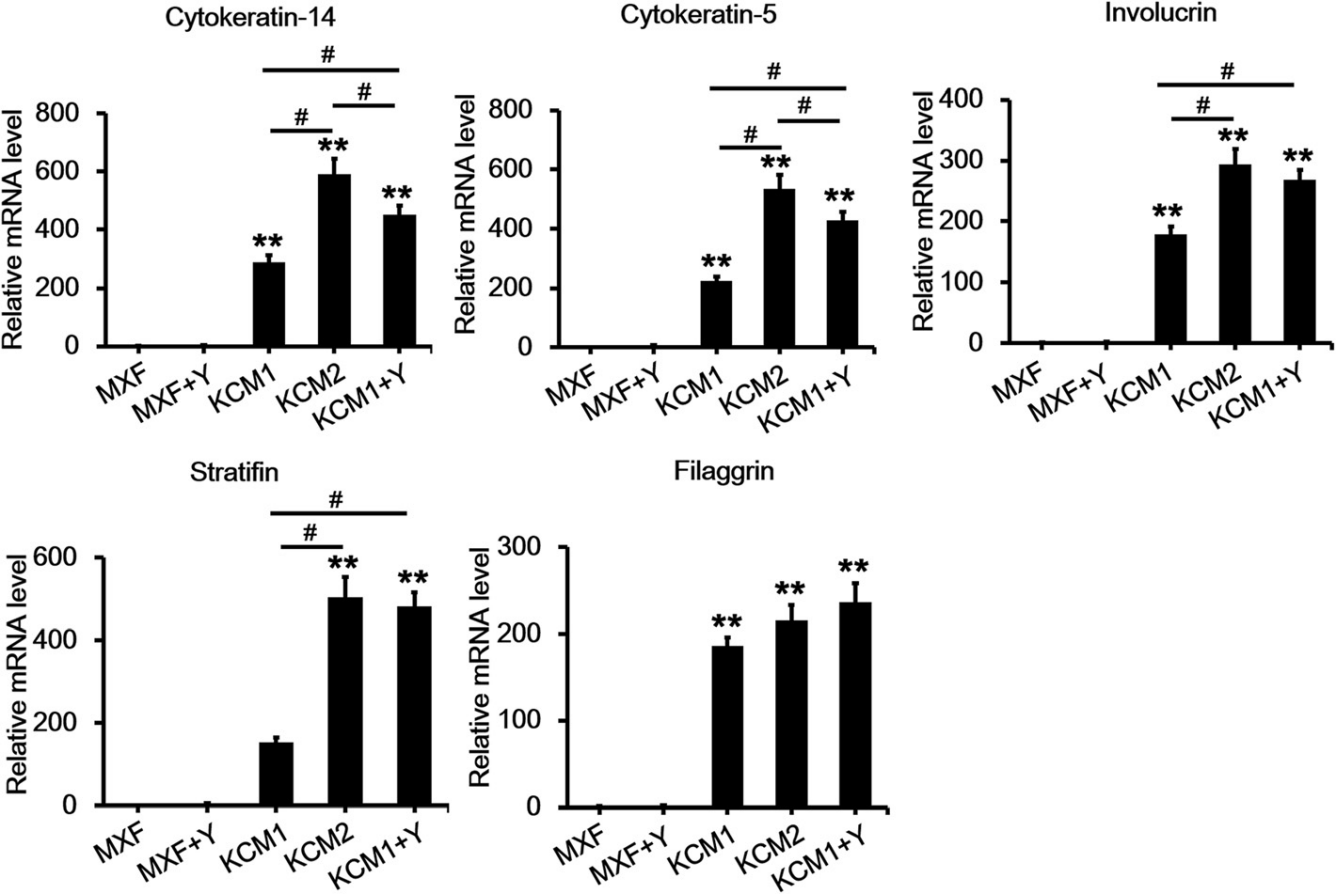
**Y-27632 can act both directly and indirectly on bone marrow mesenchymal stem cells (BMSCs) to promote their differentiation into keratinocyte-like cells.** mRNA expression of cytokeratin-5, cytokeratin-14, stratifin, involucrin, and filaggrin in BMSCs under different conditions is shown. BMSCs treated with MesenCult-XF medium were normalised to 1. Data are presented as the mean ± standard error of the mean. n = 3. **P <0.01 MXF versus keratinocyte-conditioned media, ^#^ *P* <0.05. KCM1, keratinocyte-conditioned medium collected from keratinocytes with no Y-27632 treatment; KCM2, keratinocyte-conditioned medium collected from keratinocytes treated with Y-27632; KCM1 + Y, KCM1 plus Y-27632; MXF, MesenCult-XF medium; MXF + Y, MesenCult-XF medium plus Y-27632.

Next, we investigated whether the effects on differentiation were mediated by Y-27632 itself or by factors whose secretion from keratinocytes was induced by continuous treatment with Y-27632. To further explore this issue, the following different types of media were used: MesenCult-XF medium (control) or MesenCult-XF medium plus Y-27632, KCM1, KCM2, or KCM1 + Y-27632. Compared with the control group, the expression of keratinocyte-specific markers significantly increased under all three KCM conditions, whereas Y-27632 treatment alone did not induce the expression of any keratinocyte-specific markers. Moreover, the level of each marker except filaggrin was significantly higher in KCM2-treated cells and in KCM1 + Y-27632-treated cells than that in KCM1-treated cells, and further comparison showed that the expression of cytokeratin-14 and cytokeratin-5 in the KCM2 group was higher than in the KCM1 + Y-27632 group (Figure 7). Together, these results suggest that both KCM derived from Y-27632-treated keratinocytes and Y-27632 itself can induce the differentiation of BMSCs into KLCs in our xeno-free induction system, although the former led to a higher differentiation efficiency.

## Discussion

In the present study, we successfully established a xeno-free system for the culture of keratinocytes and for the differentiation of BMSCs into KLCs. Furthermore, we provided strong evidence that BMSCs can differentiate into KLCs under this condition and that the inhibition of ROCK by Y-27632 can lead to the inhibition of keratinocyte senescence and to the promotion of induction efficiency.

As the traditional and most well-characterised source of human MSCs, BMSCs are increasingly being used in clinical applications because of the availability of a large amount of data regarding their biological characteristics and their safety in clinical studies [11]. Currently, most of the procedures for BMSC expansion involve the use of bovine serum-containing medium for culturing the cells and porcine-derived trypsin for dissociating the cells. The use of these two components raises concerns for the transmission of unknown zoonoses and for the induction of immunological reactions after clinical transplantation. Thus, the development of xeno-free, serum-free medium for BMSCs has become a high priority. In the present study, MesenCult-XF medium, which is a defined serum- and xeno-free medium, was used for the isolation and cultivation of BMSCs. Our results showed that the growth and characteristics of BMSCs that are grown under this culture condition are consistent with those in xeno-containing medium as reported previously [11]. Similarly, DKSFM was used to obtain xeno-free conditioned medium for the culturing of human primary keratinocytes. Thus, we were able to successfully avoid the potential contamination derived from animal serum and feeder cells in conventional keratinocyte medium.

The *in vitro* differentiation of BMSCs into cardiomyocytes, neurons, epithelial cells, myofibroblasts, and other lineages has been reported by using different induction protocols [25-28]. However, no previous reports examined the differentiation of BMSCs into keratinocytes. Our present study provides new evidence that human BMSCs can also differentiate into KLCs in a xeno-free induction system *in vitro*. This new finding is consistent with the concept that BMSCs can differentiate into cells from all three embryonic germ layers and with a previous report that BMSCs can differentiate into keratinocytes *in vivo* [29]. Furthermore, our findings support the possibility of cultivating abundant keratinocytes *in vitro* for potential clinical applications and for basic scientific research.

ROCK is a downstream effector of the Rho GTPases, which control a variety of cellular processes, including cytoskeletal dynamics, cell polarity, membrane transport, and gene expression [2,17,30]. Y-27632, which is a known inhibitor of ROCK, has been shown to affect the differentiation and proliferation of multiple types of stem cells [20,31,32]. In a recent study, we showed that the life span of primary human keratinocytes was prolonged significantly in cultures treated with the ROCK inhibitor Y-27632 in DKSFM. This result is consistent with previous findings in keratinocytes [16,21]. However, this result differs from a previous report that Y-27632 could enable human primary keratinocytes to efficiently bypass senescence and become immortal cells [15,16]. This disparity may be due to differences in the protocols used for the culture of keratinocytes. More intriguingly, we further demonstrated that all three groups of KCMs (KCM1, KCM2, and KCM1 + Y-27632), but not Y-27632 alone, were able to induce the differentiation of BMSCs into KLCs when added to the MesenCult-XF medium. These results suggested that KCM was necessary for the differentiation of BMSCs into KLCs and corroborated a previous finding that conditioned medium contains secreted growth factors and small molecules that are able to activate BMSC differentiation [7,24]. Furthermore, the efficiency of differentiation was significantly higher in KCM2-treated cells and in KCM1 + Y-27632-treated cells than in KCM1-treated cells. This result indicates that Y-27632 can act both directly and indirectly on KCM-treated BMSCs to increase their differentiation efficiency. More importantly, based on the observation that the differentiation efficiency in the KCM2 group was higher than that in the KCM1 + Y-27632 group, Y-27632 may activate keratinocytes to release critical factors and molecules that promote the differentiation of BMSCs into keratinocytes under our conditions. However, the specific molecules and mechanisms that are involved in this process remain to be determined. In conclusion, these data indicate that Y-27632 is important for the differentiation of BMSCs into keratinocytes in addition to its importance in the differentiation of BMSCs into other types of cells [33,34]. Therefore, Y-27632 may have a conserved role in the differentiation of BMSCs.

Keratinocytes are the primary cell type in the epithelial layer of the skin, which provides an essential function as a protective barrier against insults from the outside environment. Many previous studies have shown that keratinocytes can influence wound healing in the skin [35-39]. However, obtaining a sufficient amount of keratinocytes from the adult skin is difficult because of the low expansion capacity and the short life span of primary human keratinocytes [21]. Our findings that BMSCs can successfully differentiate into KLCs suggest a novel method for obtaining many keratinocytes *in vitro* and further support the potential of BMSCs as a promising therapy for skin defects. Our results also suggest that keratinocytes may play a key role in the BMSC-mediated improvement of cutaneous wound healing as reported previously [29,40-42]. Moreover, our demonstration that the ROCK inhibitor Y-27632 causes a significant increase in the efficiency of the differentiation of BMSCs into KLCs, together with a previous report that Y-27632 can facilitate the differentiation of BMSCs into neural cells [34], raises the intriguing possibility that the ROCK pathway plays a critical regulatory role during BMSC differentiation. Furthermore, these results suggest a potential approach to improve the therapeutic effect of BMSCs in patients with skin defects.

Although we demonstrated that the efficiency of the differentiation of BMSCs into KLCs was significantly higher in KCM treated with the ROCK inhibitor Y-27632, the mechanism underlying this effect remains unclear. Based on previous studies, this effect is likely mediated by Akt activation, increased MYC activity, and/or downstream target phosphorylation [15,31]. However, further studies are required to distinguish among these possibilities. Furthermore, whether the ROCK inhibitor Y-27632 can enhance the differentiation of MSCs into keratinocytes *in vivo* remains unclear.

## Conclusions

The present study demonstrated that BMSCs have the capability to differentiate into KLCs in a xeno-free induction system with additional KCM. Moreover, the ROCK inhibitor Y-27632 not only promoted keratinocyte proliferation and inhibited keratinocyte senescence but also increased BMSC differentiation efficiency. These findings further support the potential clinical application of BMSCs in skin regeneration for patients with massive skin loss or with other skin diseases and provide useful insights into the mechanisms underlying the differentiation of BMSCs into KLCs.

## Abbreviations

BMSC: bone marrow mesenchymal stem cell
BSA: bovine serum albumin
DKSFM: defined keratinocyte serum-free medium
FBS: foetal bovine serum
KCM: keratinocyte-conditioned medium
KLC: keratinocyte-like cell
mAb: monoclonal antibody
MNC: mononuclear cell
MSC: mesenchymal stem cell
PBS: phosphate-buffered saline
PCR: polymerase chain reaction
polyAb: polyclonal antibody
ROCK: Rho kinase
rpm: revolutions per minute
RT-PCR: Real-time-polymerase chain reaction
